# Tuina for shoulder pain after stroke: A protocol for systematic review and meta-analysis

**DOI:** 10.1097/MD.0000000000031828

**Published:** 2022-11-18

**Authors:** Weichen Sun, Guangcheng Ji, Longman Lu, Jiabao Sun, Haoze Guo, Yao Yao, Shan Gao, Jing Li, Jinjin Chen, Bailin Song

**Affiliations:** a Department of Acupuncture and Tuina, Changchun University of Chinese Medicine, Northeast Asia Research Institute of Traditional Chinese Medicine, Changchun, China; b Department of Rehabilitation, The Third Affiliated Hospital of Changchun University of Chinese Medicine, Changchun, China; c Beihua University, Jilin, China; d Jilin Ginseng Academy, Changchun University of Chinese Medicine, Changchun, China; e Jilin Province Federation Rehabilitation Center for the Disabled, Changchun, China.

**Keywords:** post-stroke shoulder pain, protocol, systematic review, Tuina

## Abstract

Post-stroke shoulder pain is a relatively common complication in stroke patients, with an incidence of 16% to 84% and poor outcomes with anti-inflammatory or sedative medications. This study will evaluate the results of a randomized controlled trial to determine the effectiveness and safety of Tuina in the treatment of post-stroke shoulder pain.

**Methods:**

The Chinese and English search strategies were used to search China National Knowledge Infrastructure, Chinese Scientific Journal Database, Cochrane Central Register of Controlled Trials, EMBASE, MEDLINE, PubMed, Wanfang Database, and Web of Science were used to search seven databases. All eligible studies published on or before September 15, 2022, will be selected. To improve the validity of this study, only clinical randomized controlled trials related to the use of Tuina for post-stroke shoulder pain will be included. The screening will be performed by 2 independent reviewers and data synthesis, bias analysis, subgroup analysis, and meta-analysis will be performed using RevMan (V.5.4) software.

**Results:**

The study will provide a high-quality evaluation of the effectiveness and safety of Tuina in the treatment of post-stroke shoulder pain.

**Conclusion:**

This systematic review will provide evidence to determine whether Tuina is an effective and safe intervention for treating patients with post-stroke shoulder pain.

PROSPERO registration number: CRD42022360401.

## 1. Introduction

Stroke is the second leading cause of mortality worldwide, and although mortality has been reduced by early diagnosis, lifestyle changes, and aggressive management of vascular risk factors with medication, stroke remains the leading cause of disability.^[1,2]^ Recovery of upper extremity mobility is usually poor after stroke, with approximately 85% of stroke survivors having upper extremity dysfunction. Post-stroke shoulder pain is a common complication after stroke, and the prevalence of post-stroke shoulder pain has been reported to range from 16% to 84%, depending on the age of the study population, the time of onset of post-stroke shoulder pain, the specific site of onset, and different assessment methods.^[3–5]^ The etiology of HSP is difficult to assess and mechanisms include structural damage due to glenohumeral subluxation, capsular contracture, or rotator cuff lesions, as well as risk factors associated with impaired sensation, spasticity and flaccid paralysis due to neurological damage.^[6–10]^ The symptoms are pain, numbness, burning pain or abnormal sensation in the shoulder, and the limitation of shoulder joint movement, which seriously affects the rehabilitation process and the recovery of motor function of the affected limb, causing significant emotional and psychological disorders and even depression. Common treatments in clinical practice include physical therapy, local cooling, infrared light, local anesthesia, botulinum toxin injection, and corticosteroid injection, but there is insufficient evidence to prove their effectiveness.^[11]^

In China, Tuina therapy is used as a conventional treatment for various diseases as an external treatment method, and it is similar to acupuncture therapy in mechanism. Through the body meridian points and disease-related meridian acupoints, different manipulation techniques are used for external treatment penetration, which can help to release adhesions, reduce pain, and increase pain threshold, thus contributing to rehabilitation, enhance the number of passive movements, increase range of motion, and improve joint mobility disorders.^[12,13]^ Currently, there is no high-quality systematic evaluation of Tuina for post-stroke shoulder pain, so this study will evaluate the efficacy and safety of Tuina for post-stroke shoulder pain to provide a basis for clinical decision-making.

## 2. Methods

The systematic review will be conducted by the 2015 Preferred Reporting Items for Systematic Reviews and Meta-Analyses Protocol (PRISMA-P) guidelines and the study protocol is registered on PROSPERO (registration number: CRD42022360401). As this study does not require patient recruitment and collection of personal data, an ethics statement is not required.

### 2.1. Inclusion criteria

#### 2.1.1. Types of studies.

We will include randomized controlled clinical trials and quasi-RCTs of Tuina for post-stroke shoulder pain in the treatment group. In the case of a multi-arm RCT, we will select the group that uses Tuina and another group that does not use Tuina for analysis. We will select a phase I crossover RCT in which one group uses nudging for the first time. The languages of the RCTs include English and Chinese.

#### 2.1.2. Types of participants.

Inclusion criteria: patients with a clinical diagnosis of post-stroke shoulder pain, aged less than 70 years, with a duration of illness between 1 month and 6 months, regardless of gender and severity of shoulder pain, and with a Visual analog scale score of no less than 3.

Exclusion criteria: patients with severe cardiac, hepatic, renal, or metabolic diseases, psychiatric disorders, or tumors. Acute shoulder soft tissue injury, fracture, dislocation, infectious inflammation, cervical spondylosis, rheumatoid arthritis, gout, etc. Other diseases cause pain and restriction of movement around the shoulder joint.

#### 2.1.3. Types of interventions.

We will include studies in which the intervention group received Tuina alone or in combination with routine rehabilitation treatment (manual therapy, exercise therapy, electronic biofeedback, oral medication, acupuncture, Chinese herbal medication, physical therapy, surgery, botox injections and so on or even with no treatment).

#### 2.1.4. Types of outcomes.

Primary outcomes included a Simplified Fugl–Meyer Movement Assessment. Secondary outcomes included the Visual analog scale, Range of motion of the shoulder joint, and modified Barthel index.

### 2.2. Data sources and search methods

#### 2.2.1. Electronic searches.

We will search eight databases including China National Knowledge Infrastructure, Chinese Scientific Journal Database, Cochrane Central Register of Controlled Trials, Embase, MEDLINE, PubMed, Wanfang Database, and Web of Science from inception to September 15, 2022, and eligible literature will be independently selected based on inclusion and exclusion criteria.

#### 2.2.2. Search strategy.

We will base our search on the Cochran Handbook guidance, including medical subject heading terms and variants, and will search for: post-stroke shoulder pain, PSSP, shoulder pain, stroke, post-stroke, Tuina, massage, and all related subject terms. Table 1 lists the detailed search strategy for PubMed, while the search strategy will be modified according to other different databases (Table 1).

**Table 1:** Search strategy (PubMed).

Number	Search terms
#1	“tuina”[All Fields] OR “Chineses tuina”[All Fields] OR “massage”[All Fields] OR “Chinese massage”[All Fields]OR “therapy”[All Fields] OR “manual therapy”[All Fields] OR “Chineses manipulation”[All Fields] OR “Chineses manipulative therapy”[All Fields] OR“massotherapy” [All Fields] OR “Acupressure” [All Fields] OR “Massaging”[All Fields] OR “Manipulation”[All Fields]
#2	“Stroke”[All Fields] OR “Apoplexy” [All Fields] OR “post- stroke”[All Fields] OR “poststroke”[All Fields] OR “Apoplectic” [All Fields] OR “Apoplexia” [All Fields] OR “Cerebral hemorrhage” [All Fields] OR “Ich” [All Fields] OR “Cerebrovascular accident” [All Fields] OR “Cerebrovascular disorders” [All Fields] OR “Cerebral Embolism” [All Fields] OR “Brain embolism” [All Fields] OR “Embolic stroke” [All Fields] OR “Cerebral infarct”
#3	Hemiplegic Shoulder Pain”[All Fields] OR “Post-stroke shoulder pain” ”[All Fields] OR “shoulder pain after stroke ”[All Fields] OR shoulder pain”[All Fields] OR “ Shoulder-hand syndrome”[All Fields]
#4	#2 AND #3
#5	“randomized controlled trial” [Publication Type] OR “controlled clinical trial” [Publication Type] OR “Single-Blind Method” [Text Word] OR “Double-Blind Method” [Text Word] OR “random allocation” [Text Word] OR “allocation” [Text Word] OR “RCT” [Text Word]
#6	#1 AND #4 AND #5

### 2.3. Data extraction and management

**Figure 1.**
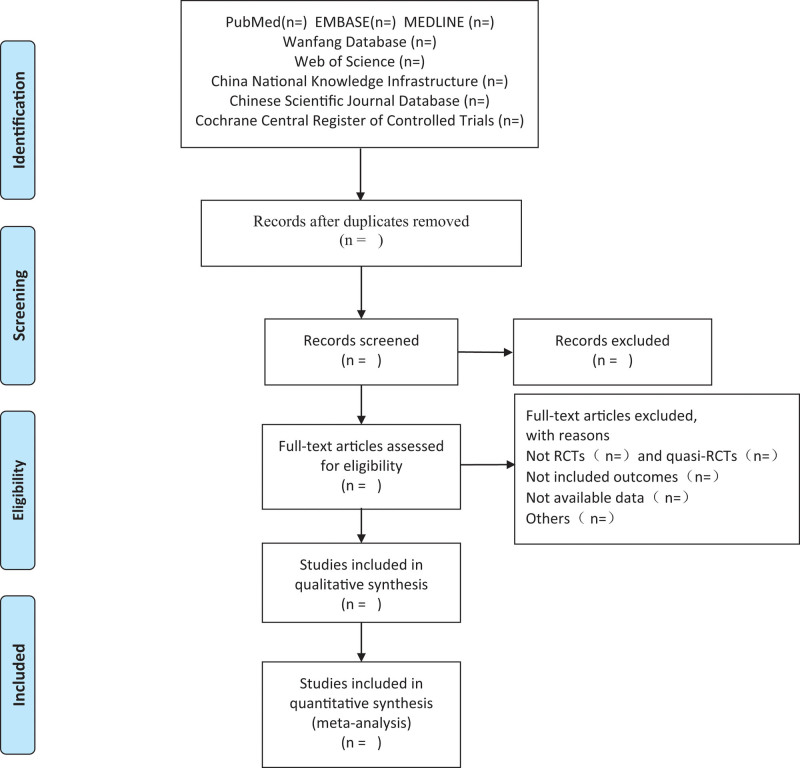
The screening process. RCT = randomized controlled trial.

Two researchers will independently select eligible literature based on inclusion and exclusion criteria, based on article title, keywords, abstract, and if the relevant content is not clear enough to read the full text to confirm if it should be included, and we will try to contact the authors when data are missing from the literature. If we were unable to obtain complete data, then the study would be excluded. We also excluded non-controlled studies, non-randomized studies, studies with inconsistent evaluation criteria, or similar data, and extracted the following information: general information (journal information, authors, publication date, language), patient information (type of stroke, time of onset, duration, sex, age), study design information (sample size, experimental group, intervention method, intervention duration), and trial results. If any discrepancy occurs during the screening process, a third investigator will intervene. The screening process is shown in Figure 1.

### 2.4. Quality assessment

Two researchers independently assessed the quality of the trials in the selected literature for risk of bias according to the Cochrane systematic review manual. Six main aspects were assessed: the pattern of random sequence generation, whether allocation concealment was used, whether subjects and intervention providers were blinded, whether outcome assessors were blinded, whether outcome data were complete, and whether outcome reporting and other sources of bias were selected. The risk of bias levels was categorized as low, high, or unclear. The evaluation process was adjudicated by a third investigator in cases where there was a need for agreement and uncertainty about the risk level.

### 2.5. Treatment effect measures

To assess the effect of Tuina therapy for post-stroke shoulder pain, our final measured continuous data were expressed as mean differences, and 95% confidence intervals were chosen to show the effect.

### 2.6. Assessment of heterogeneity

If there was no significant heterogeneity between studies (*I*^2^ < 50%), a fixed-effects model would be used for assessment. If there is significant heterogeneity (*I*^2^ > 50%), a random effects model will be used for assessment.

### 2.7. Assessment of reporting bias

In cases where more than 10 trials meet the trial criteria, we will use the Cochrane Collaboration’s Revman 5.4 software to produce funnel plots to assess bias.

### 2.8. Data synthesis

Data synthesis will be performed using RevMan V.5.4. Results are expressed as risk ratios and standardized or weighted mean differences for continuous data. The methodology is as follows: if the *I*^2^ test is less than 50%, a fixed-effects model is used for data synthesis. If the *I*^2^ test is between 50% to 75%, a random-effects model is used for data synthesis. If the *I*^2^ test is >75%, we investigate possible causes from a clinical and methodological point of view and perform subgroup analysis. If the data cannot be synthesized, we provide descriptive analysis to address this issue.

### 2.9. Analysis of subgroups or subsets

In cases of high heterogeneity, we will perform subgroup analysis to determine the source of heterogeneity. We will perform subgroup analysis also for potential factors such as gender, different duration of sessions, different types of Tuina practices, and different types of controls when the data are complete.

### 2.10. Sensitivity analysis

The sensitivity analysis focuses on the impact of sample size, methodological quality, and missing data on the study and analyzes the robustness of the overall study results.

### 2.11. Grading of evidence quality

We will use Grade^[14]^ to assess the credibility of the data of interest.

## 3. Discussion

In clinical practice, shoulder pain sometimes appears a few days after the stroke, and some stroke patients develop shoulder pain within 6 months after the stroke, and although some patients’ shoulder pain can be relieved by treatment within 8 to 12 weeks of onset, some patients with chronic shoulder pain still have shoulder pain 1 year after the stroke.^[15–17]^ The main factors affecting the improvement of shoulder pain are the occurrence of post-stroke depression, reduced daily use of the arm, lack of timely rehabilitation interventions, and poor quality of life with a heavy family burden.^[5]^ However, post-stroke shoulder pain does not heal spontaneously, and over time, patients with chronic shoulder pain will face long treatment cycles, slow recovery, and a high financial burden.^[18]^ Studies have shown that shoulder pain reduces the quality of life 12 months after acute stroke.^[19]^ A secondary analysis study confirmed^[20]^ that post-stroke shoulder pain is beneficial if early intervention is provided.

External treatments for post-stroke shoulder pain have become increasingly popular among patients in recent years in clinical practice, but with the diversity of treatments, the effectiveness of treatment lacks high-quality data indicators to assess. A meta-analysis indicated^[21]^ that botulinum toxin injections are more effective than steroid and placebo treatments, but they are expensive and require physicians to consider the patient’s financial situation for treatment. Tuina therapy is safe and easy to perform, has low treatment costs, and has no side effects, so the effectiveness of Tuina for post-stroke shoulder pain will be validated with high-quality data, hopefully providing a basis for clinical decision-making. We will also consider several issues that need attention in systematic review and meta-analysis. A comprehensive search of the literature will be conducted, but it cannot be fully confirmed that all protocol-related RCT studies were included, and some literature published in languages other than English and Chinese will be excluded. We will interpret these results with caution if the number of included studies and patients is small, as well as trials that use a mix of treatments with multiple approaches.

## Author contributions

**Data curation:** Weichen Sun, Shan Gao.

**Formal analysis:** Jing Li, Jinjin Chen.

**Funding acquisition:** Bailin Song.

**Investigation:** Haoze Guo, Yao Yao.

**Methodology:** Jiabao Sun.

**Validation:** Guangcheng Ji.

**Writing – original draft:** Weichen Sun, Longman Lu.

**Writing – review & editing:** Guangcheng Ji, Bailin Song.
